# Current Only-Based Fault Diagnosis Method for Industrial Robot Control Cables

**DOI:** 10.3390/s22051917

**Published:** 2022-03-01

**Authors:** Heonkook Kim, Hojin Lee, Sang Woo Kim

**Affiliations:** 1 Department of Electrical Engineering, Pohang University of Science and Technology, 77 Cheongam-Ro, Nam-Gu, Pohang 37673, Korea; kimhk85@postech.ac.kr (H.K.); suvvus@postech.edu (H.L.); 2 Hyundai Robotics Co., Ltd., 50, Techno Sunhwan-ro 3-gil, Yuga, Dalseong-gun, Daegu 43022, Korea

**Keywords:** autoencoder, control and instrumentation cable, fault diagnosis, industrial robot, novelty detection, soft fault

## Abstract

With the growth of factory automation, deep learning-based methods have become popular diagnostic tools because they can extract features automatically and diagnose faults under various fault conditions. Among these methods, a novelty detection approach is useful if the fault dataset is imbalanced and impossible reproduce perfectly in a laboratory. This study proposes a novelty detection-based soft fault-diagnosis method for control cables using only currents flowing through the cables. The proposed algorithm uses three-phase currents to calculate the sum and ratios of currents, which are used as inputs to the diagnosis network to detect novelties caused by soft faults. Autoencoder architecture is adopted to detect novelties and calculate anomaly scores for the inputs. Applying a moving average filter to anomaly scores, a threshold is defined, by which soft faults can be properly diagnosed under environmental disturbances. The proposed method is evaluated in 11 fault scenarios. The datasets for each scenario are collected when an industrial robot is working. To induce soft fault conditions, the conductor and its insulator in the cable are damaged gradually according to the scenarios. Experiments demonstrate that the proposed method accurately diagnoses soft faults under various operating conditions and degrees of fault severity.

## 1. Introduction

Control and instrumentation (C&I) cables are utilized in a wide range of industrial applications, including nuclear power plants [1], ship power systems [2], vehicles [3], and factory automation [4], owing to their vital role in the control of motors and instrumentation of sensors. To maintain the stability of automated systems in various applications, monitoring the health status of the C&I cable is crucial. Early and accurate diagnosis of faults reduces unwanted system downtime and improves the system reliability, resulting in the maximization of productivity. In modern automated manufacturing processes, a single fault in an industrial machine could cause an entire production line stoppage because the safety signals of machines are shared and controlled by a process control unit. Researchers have studied the faults from industrial machines. A diagnosis method based on an artificial neural network using most salient features has shown reliability in diagnosing [5]. In the other hand, the modern robust control studies have shown that certain types of robot faults can be overcome with compensation of perturbations [6].

Cable faults comprise hard faults and soft faults. Hard faults include open circuit and short circuit, and soft faults are characterized by small impedance changes. A local modification of the cable (soft fault) due to a harsh environment could be transformed into hard fault by subsequent partial damage of the components, such as the cable conductors, coatings, and shield [7]. The worst possible case occurs when the machine is still working without any indication of cable faults, while the cable is partially damaged. This is a common practical soft-fault problem that occurs in automated factories. Therefore, timely diagnosis of a soft fault before it becomes severe is important to prevent further damage to automated machines in production lines.

Conventional cable-condition assessment can be divided into electrical techniques, such as tan delta (TD) and partial discharge (PD) techniques; mechanical techniques, such as elongation at break technique; and chemical techniques, such as oxidation induction time (OIT) technique. However, these techniques can only be implemented in either destructive or laboratory environments. Moreover, these methods do not facilitate online diagnosis of operating cables because the machine must be turned off to disconnect the cables.

Until now, the efficiency of reflectometry-based methods for cable diagnostics has been demonstrated [8,9,10,11,12,13,14]. In particular, time-frequency domain reflectometry (TFDR) [12,13] proves the robustness of fault diagnosis in a variety of conditions because it utilizes information in time and frequency domains. Although these methods diagnose hard faults effectively owing to its huge reflection, their diagnostic accuracy for small anomalies is not always effective because of the physical difficulties, such as the ambiguous response of the incident signals [7]. In addition, these reflectometry-based methods require additional equipment, such as signal generators and sensors, for the diagnosis. Moreover, these methods require prior domain knowledge and manual feature extraction during the test and analysis processes.

Online detection of cable faults has been studied for various applications, including aircraft [14] and power distribution networks [15]. A method based on spread-spectrum time-domain reflectometry (SSTDR) has been proposed and proven to be a suitable fault-diagnosis technique for online detection in the aircraft data bus [14]. However, this method is not suitable for the detection of soft faults in motor control cables owing to the high current in a motor. In addition, a method for the diagnosis of cable-sheath faults using the metal sheath current in power distribution networks was proposed in [15]. This approach allows online fault diagnosis of power distribution networks. However, it utilizes link boxes of the high voltage cables, which are not installed in automated factories. Moreover, this method is complex for the direct detection of soft faults in a cable conductor, which is surrounded by the cable sheath.

The control cable used in automated machines, such as an industrial robot, differs from the cable used in power plants in that the frequency, amplitude, and phase sequence vary according to the robot command and are affected by operating conditions, such as power regeneration. Therefore, existing studies, which assume constant conditions, are not applicable to this field [16]. Moreover, the length of transmission lines in power plants is several kilometers, whereas those in smart factories are often extremely short according to the factory layout. Existing traditional and reflectometry-based methods do not sufficiently address faults in very short cables from factory automation domains, such as vehicle manufacturing lines and smart factories.

In addition, because automated machines in the factory floor are not easily accessible, additional equipment is not preferable when conducting diagnosis. Therefore, all the diagnosis methods that require additional equipment [8,9,10,11,12,13,14,15] are not preferable. In addition, the automation machines in manufacturing lines work round the clock; therefore, using an offline diagnostic approach with no online diagnostic capability is not preferable. More importantly, destructive and intrusive diagnostic approaches, such as mechanical and chemical methods, are not applicable under this condition because the automated machines still need to work to attain the manufacturing target and are repaired only according to the scheduled maintenance time.

Unlike hard faults, soft faults are of various patterns. Thus, soft faults cannot be perfectly reproduced in laboratories because the length, cross-sectional area, and the shape of the fault is indefinitely varying. In particular, the fault length is sometimes overlooked although it has a strong impact on the diagnostic results [17]. In addition, collecting fault data, specifically soft-fault data, is often very difficult because the machine can work even with the unhealthy (soft faulty) cables, without any indication of the fault. Thus, existing works, which artificially simulate faults using variable resistors and establish a diagnosis model accordingly, are difficult to be applied in the field because fault data are rare, and the simulated faults are not significantly realistic. To compensate for these short comings, a novelty detection-based approach is an excellent alternative. Novelty detection with numerous normal-operation data can be established easily and is practically a more realistic method of diagnosing soft faults.

Novelty detection is the process of finding data points that do not belong to the normal data [18]. There are many applications of novelty-detection, such as medical anomaly detection, video surveillance, and industrial anomaly detection [19]. Novelties were successfully detected using reconstruction errors a long time ago in [20]. A reconstruction error is the distance between the network input and the reconstructed input. Autoencoders (AE) encode the input into the reduced representation and then reconstruct the input by learning how to reconstruct the data back from the reduced representation as close to the original input as possible. The novelty detection-based diagnosis method assumes that novelties, which represent faults, cannot be reconstructed perfectly from the reduced representations [21]. Therefore, the data points with high-reconstruction errors can be regarded as novelties.

This article proposes a novel soft-fault diagnosis algorithm that diagnoses the faults in the control cable of industrial robots using an encoder-decoder structure with the reconstruction along projection pathway (RaPP) module [22]. The RaPP module compares the input and its reconstruction, not only in input space, but also in the hidden spaces in the trained network. Therefore, the RaPP supports the decision process by providing additional information, such as pairs of hidden activation values from projecting input and hidden reconstructions along the projection pathway of AE. The network receives the sum of three phase currents and the ratios of squared single-phase currents (SCs) over the summation of squared three-phase current (SSC) as inputs. The experiment validates that the proposed diagnosis model successfully diagnoses the fault occurrence even under varying fault and operating conditions. The major contributions of this study are as follows:

(1) Only three-phase currents are used. Therefore, additional sensors, signal generators, and signal inducing devices are not required, making the method cost efficient, whereas the other methods [8,9,10,11,12,13,14] need additional devices to generate a reference signal and to inject the reference signal into target cables. For example, an arbitrary waveform generator is used to create the designed reference signal in [8,9,10,12,13,14]. In [11], a network analyzer is used to inject sinusoidal waves into the target cable and to measure the reflected waveforms. For signal injection, an inductive coupling device is used in [9], whereas others [8,10,12,13,14] utilize three-way connectors, such as T-connectors, and directional bridges.
(2) No manual feature extraction with prior knowledge and mathematical values of cable parameters is required, whereas reflectometry based methods need physical cable parameters, such as the information of cable length, to design an incident signal by adjusting signal bandwidth, center frequency, and time duration. In addition, surrounding noise conditions, for example 60 Hz harmonic noise in power plant, need to be analyzed before building diagnostic model to restore the reflected incident signal, which is distorted by attenuation and dispersion during propagating through target cables, using compensate filters and notch filters. Furthermore, the AE structure encodes the input data into the reduced representation automatically during learning process, whereas existing works need to analyze the selected features of a resultant signal in time and frequency domain based on prior domain knowledge.
(3) Unlike the reflectometry-based methods with blind spot issues, which occur because the incident and reflected signals may overlap for excessively short cables with falsely selected signal bandwidth and measurement instruments [23], whereas the proposed fault diagnosis is applicable to very short cables because the transmitted signal is utilized for the diagnosis.
(4) Online cable-fault diagnosis is possible using the proposed method even when a machine is operating in production lines, whereas traditional methods such as TD, PD and OIT, and recent methods such as TFDR and SSTDR cannot guarantee its diagnostic accuracy under varying conditions, such as varying frequency, amplitude, and phases when machine is working.
(5) The diagnosis method does not require numerous fault data because it is based on an unsupervised learning model, which requires only normal operation data by assuming unimodal normality. Thus, it is easily applicable in the industry, whereas other methods usually based on fault dataset to build diagnosis model by analyzing and learning the dataset, and utilized variable resistors and MATLAB simulations to artificially reproduce fault conditions.
(6) The proposed diagnosis method can be applied to various types of control cables, regardless of the conductor thickness, insulator material of cable, and other cable parameters because the method only utilizes three-phase currents through a control cable, whereas other diagnosis models need to be rebuilt when target cable is changed because the model parameters, such as cable length, velocity of propagation and bandwidth of signal etc., need to be changed according to the new target cable.

The rest of this article is organized as follows. The severity of soft faults and their effects are presented in Section 2. In Section 3, we propose a fault diagnosis model. Section 4 presents the experimental results. Finally, Section 5 concludes this article.

## 2. Soft Faults and Features in Control Cable

Soft faults are the faults associated with small impedance changes because of a local modification in the cable, such as a local modification in a part of conductor or insulation layer due to harsh environment. The soft-fault resistance *R_f_* directly represents the damage severity of the conductor because the it is inversely proportional to the reduced cross-sectional area *S_f_* of the faulty conductor; the relation can be expressed as follows:

(1) $$S_{f}=\alpha \;S_{o},\;0<\alpha \leq 1$$

(2) $$R_{o}=\rho \frac{l}{s_{o}}$$

(3) $$R_{f}=\rho \frac{l}{s_{f}}$$

(4) $$R_{f}=\rho \frac{l}{\alpha \;s_{o}}=\frac{1}{\alpha }\;R_{o}$$

where *S_o_* is the cross-sectional area of the normal cable, and *S_f_* is the remaining cross- sectional area after the damage is induced. *α* indicates the degree of damage according to the cross-sectional area of the damaged conductor in the cable. $R_{o}$ is the resistance of the normal cable, and $R_{f}$ is the fault resistance of the faulty cable, where ρ is resistivity of the conductor material and l is the length of the conductor. The value of *α* is one for a healthy cable and less than one for a faulty cable. For an open and short circuit, *α* is respectively zero and greater than one. In this study, we focused on soft faults and healthy cases, i.e., 0<α≤1. Therefore, *α* = 0 and *α* > 1 cases were not considered in this study.

The currents flowing through the fault location are affected by the damaged conductor as well as the damaged insulator due to fault resistance *R_f_* and leakage current flowing through the damaged insulation layer resulting in a power-circuit imbalance. Three-phase currents will be utilized to diagnose faults for the study because the type of the motor in the robot joint is 3-phase AC servo motor, which means that the control cable between the controller and the motor has a 3-phase power network. In addition, most of industrial controllers measure and use currents to control subjects. Therefore, the current information is easily accessible in most cases without additional equipment. The three-phase currents flowing in a C&I cable can be expressed as follows:

*i_a_* (*t*) = *I_ma_*
*cos* (*ωt* + *ϕ_a_*) (5)

*i_b_* (*t*) = *I_mb_*
*cos* (*ωt* + *ϕ_b_* − 120°) (6)

*i_c_* (*t*) = *I_mc_*
*cos* (*ωt* + *ϕ_c_* + 120°) (7)

where *I_ma_*, *I_mb_*, and *I_mc_* are the amplitude of the three-phase currents and *ϕ_a_*, *ϕ_b_*, and *ϕ_c_* are the corresponding phase angles. The current ratios of each single-phase current to the total current directly reflect the imbalance in the faulty control network. The current ratio *P_j_* is defined as follows:

*SC_j_* = *i_j_*^2^, *j* = *a*, *b*, *c*

*SSC* = *i_a_*^2^ + *i_b_*^2^ + *i_c_*^2^ (8)

*P_j_* = *SC_j_/SSC*,

where *j* represents either of the phases *a*, *b*, and *c*. The squared current ratio *P_j_* compares the proportion of each single-phase current (*SC_j_*) with the total three-phase currents (*SSC*). Consequently, it reflects the faulty power network having a fault resistance *R_f_*. The shrunk cross-sectional area *S_f_* in a single phase causes a phase imbalance and has harmful effects, such as power loss, additional heating, and energy decrease of the equipment [24]. The asymmetry in phase impedance leads to the imbalance in the phase, leading to the imbalance of the three-phase currents.

**Figure 1:**
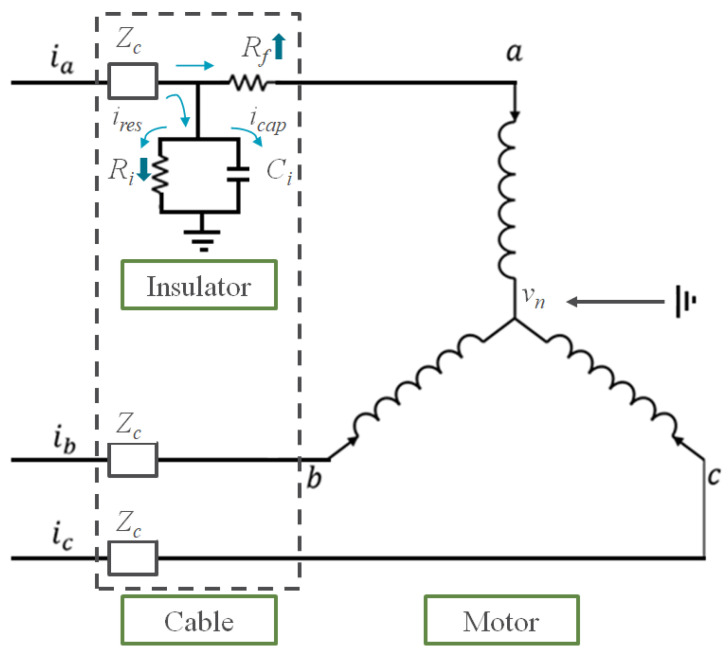
Equivalent circuit model of the faulty cable.

An equivalent circuit model under a soft fault in phase *a* of the three-phase control cable is shown in Figure 1. The control cable is connected to the permanent magnet motor of the robot joint. Figure 1 also shows a soft fault being modeled as an additional resistor *R_f_*, which represents the reduced cross-sectional area of the conductor. *Z_c_* represents the characteristic impedance of the healthy conductor. The insulator of the conductor is modelled by *R_i_* and *C_i_* in parallel because the dielectric response of the insulation can be described by its capacitive characteristic to store charge and its conductive characteristic to conduct charge [25]. The magnitude of the leakage current *i_res_* is smaller than that of the capacitive current *i_cap_* in a healthy wire. However, *i_res_* increases when the conductor is damaged (*R_f_* increases), and the insulator is damaged (*R_i_* decreases). Therefore, the vector summation of the three-phase currents is non zero because of the leakage current, which is caused by the unhealthy wire. Thus, the vector summation of the three phase currents at the wire input stage is used as a feature in this study and is expressed as follows:

(9) $$i_{sum}=i_{a}+i_{b}+i_{c}$$

The magnitude of $i_{sum}$ is zero for healthy wires although it is non-zero when soft faults are induced in the wires.

In addition, the imbalance in the three-phase currents can be described by a negative-sequence current (NSC), which can be calculated using Equation (10).

(10) $$i_{NS}=\frac{1}{3}\left(i_{a}+a^{2}i_{b}+ai_{c}\right)$$

where $i_{NS}\;$ is the NSC of the three-phase currents. $a={\;e}^{j\frac{2}{3}\pi }$ is an operator for phase rotation. NSC is a suitable parameter to diagnose the imbalance in the control network because NSC is present in addition to positive-sequence current if an imbalance exists; for example, if loading or transmission lines are unbalanced. Although the healthy-motor and control-cable assembly has a small value of NSC owing to the inherent asymmetry of the control network, the value of NSC fluctuates remarkably in the event of a soft fault. The worse the fault, the greater the imbalance in the three-phase currents. However, the motor in the robot joint rotates at a varying frequency (*ω*), varying amplitude (*I_m_*), and varying phase (*ϕ*) according to the programmed robot-motion command. Therefore, NSC cannot be a reliable tool because its value is unstable under varying operating conditions; for instance, when the motor rotates in reverse direction after a forward rotation to move the robot manipulator up and down. On the other hand, the vector summation of the three-phase currents $i_{sum}\;$ at the wire input stage is independent of these changes because no phase-rotation operator is involved. Moreover, *P_j_* is also independent of this change because it refers to the current ratios, which is just a simple number related to each phase’s current magnitude.

The analysis presented so far regarding the soft faults shows that the fault in the control cable is closely related to $i_{sum}$ and *P_j_*. This suggests that a faulty condition can be detected directly using these features. Therefore, we propose a deep AE structure to detect novelty, which is the imbalance in phase currents resulting from the faulty control network having fault resistance *R_f_*, by learning the normal operation data, with $i_{sum}$ and *P_j_* as inputs. The inputs are consistent under normal operation but experience a significant change in a faulty situation.

## 3. Diagnosis System Structure

The proposed diagnosis method comprises anomaly detection and isolation of cable fault by generating a maintenance call. An anomaly detection-based approach is adopted because the fault information is significantly lesser than the normal-operation information in industrial sites, such as automotive manufacturing plants and chemical manufacturing plants, resulting in an imbalanced dataset. Because of the imbalanced dataset, previous studies artificially reproduced fault conditions in laboratories using variable resistors and MATLAB simulations. First, we tried to use these methods to diagnose the cable-fault problems in our robot manipulators installed in the customer’s factory. However, the previous studies were not effective for the real fault conditions because the faults reproduced in the laboratory or simulation were never same as real ones that occur in factories; hence, the methods diagnosing the fictitious faults were not applicable to the practical problems.

Unlike hard faults, such as open and short circuits, soft faults can have an infinite variety of fault conditions because they can vary according to the fault length, cross-sectional area of the conductor, and even the condition of the conductor insulation layer. Therefore, the anomaly detection approach, which utilizes only normal operation dataset to learn normal conditions and isolate fault conditions, is considered as proper for soft fault diagnosis. AE is utilized for anomaly detection, and an RaPP module is added to improve the anomaly-scoring metric, using additional information in the hidden space of the trained AE.

Owing to the features, $i_{sum}$ and *P_j_*, defined in Section 2, the anomaly detection of a live cable delivering a fluctuating current to the motor is possible because these features are stable even when rotational speed, load torque, and (phase) direction of robot joint are changing. These features can only fluctuate if the power network in control cables is imbalanced. Therefore, this anomaly condition can be detected and isolated by the proposed soft-fault diagnosis under the changing operating conditions.

The architecture of the proposed model comprises a signal processing module, AE network with RaPP to detect anomalies, and anomaly monitoring module. The AE adopted in this study is the adversarial autoencoder (AAE). The advantage of AAE over other AEs, such as standard AE and variational autoencoder (VAE), is that the encoding distribution becomes similar to the prior distribution in the adversarial training process. We compared the anomaly detection capability of AAE with and without RaPP scoring technique, and the results are presented in the experimental results section.

### 3.1. Overall Structure of Diagnosis System

**Figure 2:**
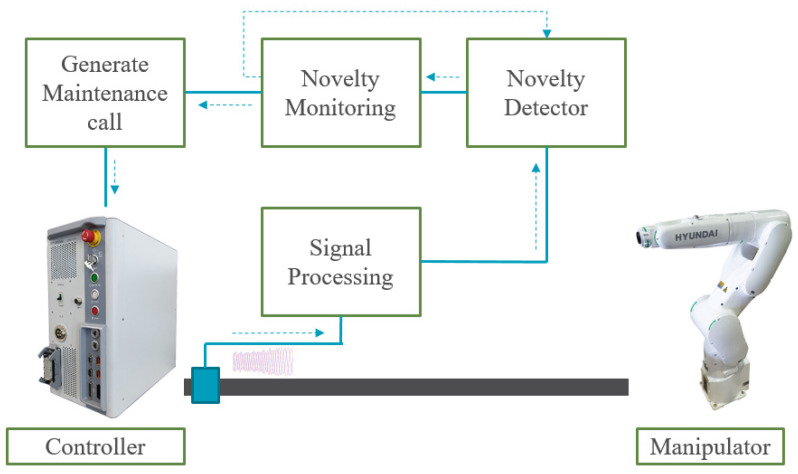
Overall structure of the proposed diagnosis system.

The overall structure of the fault-diagnosis system is shown in Figure 2. Windows of length 9000 were selected from the measured three-phase current data to construct the training dataset. The four features—*P_a_*, *P_b_*, *P_c_*, *I_sum rms_*- were obtained by calculating each window using Equations (8) and (9) and passed as inputs to the encoder-decoder network. The features were properly normalized before being used as the inputs.

The diagnosis of soft faults comprises five steps. The first step is to train the AAE only with healthy samples. After the AAE is trained, the next step is to obtain pairs of activation values by projecting the input and its reconstructed input into the trained AAE. As a result, the novelties can be identified by calculating the anomaly scores. The monitoring module checks the anomaly score per given window and moving average is applied to these values for using it as a fault indicator. If the indicator satisfies the alarm condition, the monitoring module causes the system alarm to call for maintenance 

### 3.2. AAE

**Figure 3:**
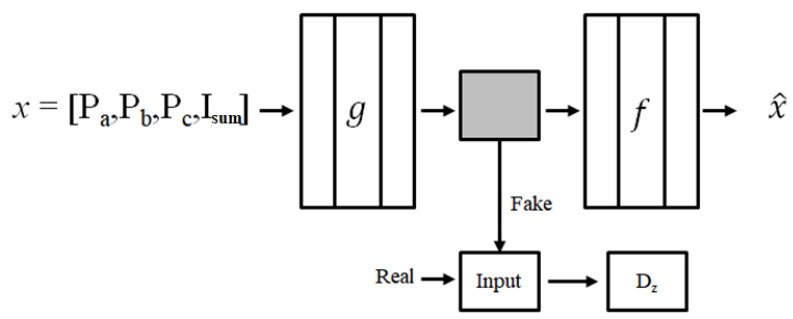
Graphical illustration of the AAE architecture used in this study. The encoder g compresses the input x and the decoder f reconstructs the input $\hat{x}$. *D_z_* acts as a discriminator through learning process.

An AAE *A* consists of an encoder g and a decoder *f*. The encoder compresses the input and reduces the dimension of the input, whereas the decoder is its inverse mapping, i.e., it reconstructs the input back from the reduced dimension as A=f◦g. The chosen features, such as *P_a_*, *P_b_*, *P_c_*, *I_sum_*, are the inputs of proposed network. The encoder encodes the input features into a reduced representation, i.e., a latent code. Thus, the encoder acts as a latent-code generator and attempts to mislead the discriminator *D_z_* into believing that the latent code is from the normal distribution. The role of *D_z_* is distinguishing whether a given sample is generated by the latent code of the AAE or a random vector sample from the normal distribution. The latent code has similar distributions to the prior distribution along the training process. Figure 3 presents a graphical illustration of the architecture.

### 3.3. RaPP

Although AAE itself has shown promising results in novelty detection, the reconstruction error alone does not fully exploit the information provided by a trained AAE. Thus, the accuracy can potentially be improved using the information in its deep architectures.

RaPP process supports the decision process by comparing not only the input and the reconstructed input, but also the hidden activations and the corresponding reconstruction in the hidden space. The hidden reconstructions can be computed indirectly by feeding the reconstructed input to the trained AAE again because the corresponding hidden reconstruction is equivalent to the hidden activation of the reconstructed input [22]. Therefore, the hidden reconstruction can be computed by feeding each of $\hat{x}$ to *g*, where $\hat{x}$ is the reconstructed input from the trained AAE whose input x is given according to $\hat{x}=A\left(x\right)$. Let *ℓ* be the number of hidden layers in *g* and *f*, where $g=g_{\ell }◦\ldots ◦g_{2}◦g_{1}$ and $f=f_{1}◦f_{2}◦\ldots ◦f_{\ell }.$ The partial computation of *g* is as follows:

(11) $$g_{:i}=g_{i}◦\ldots ◦g_{1}$$

If x and $\hat{x}$ are fed into *A*, then pairs ($h_{i}$, ${\hat{h}}_{i}$) of their hidden representations are obtained as follows:

(12) $$h_{i}\left(x\right)=g_{:i}\left(x\right)$$

(13) $${\hat{h}}_{i}\left(x\right)=g_{:i}\left(\hat{x}\right)=g_{:i}\left(A\left(x\right)\right)$$

Finally, the novelty score can be obtained using these two hidden activations, ($h_{i}\left(x\right)$, ${\hat{h}}_{i}\left(x\right)$). To calculate the score, RaPP adopts the normalized aggregation along pathway (*s_NAP_*) metric [22]:

(14) $$s_{NAP}\;\left(x\right)=||\;{\left(d\left(x\right)-µx\right)}^{⊤}\;V\Sigma^{-1}-1|{|}_{2}^{2}$$

where *d*(*x*) = h(x) − $\hat{h}\left(x\right)$, *X* is a given training set, and D is a matrix whose *i*-th row corresponds to *d*(*x_i_*) for *x_i_* ∈ *X.* To normalize the distance, D̅ = U*ΣV*^⊤^ needs to be computed. This metric considers the properties of hidden spaces and distance distribution of pairs from Equations (12) and (13) to capture clear patterns.

### 3.4. Training and Testing Procedure

The purpose of training the AAE is to minimize the difference between its input *x* and output *A*(*x*). The latent code that the encoder *g* constitutes provides a more useful representation for diagnosis than the input space. First, the AAE was trained on normal data samples. Subsequently, a test sample *x* was loaded to the trained AAE to generate the reconstructed input A(x). The novelty of the test sample *x* was measured by the following reconstruction error ε formula:

(15) $$\varepsilon ={\left|\left|x-A\left(x\right)\right|\right|}_{2}$$

The test sample *x* can be identified as novel when the error ε(*x*) increases, this means that *x* is farther from the manifold described by the AAE. To improve the result, we adopted an RaPP technique to obtain the novelty score *s_NAP_*. With this novelty score, we evaluated the diagnostic accuracy using the area under the receiver operating characteristic curve (AUROC) [26]. In the experimental results section, the reconstruction error ε(*x*) and *s_NAP_* are listed together to compare the results.

We trained the network with only healthy samples and used both healthy and faulty samples during the testing. Half of the test datasets were collected from the healthy cable, and the other half were collected from the faulty cable. We assumed unimodal normality because we selected one healthy class for normality, and the other classes, that is, 4 cut–28 cut classes were selected as novelty, as shown in Table 1.

The proposed diagnosis model was implemented in the Pytorch framework [27]. Stochastic gradient descent with Adam optimizer [28] was used for minibatches of size 20. Binary cross entropy was used as a loss function with the reduction of summation. The learning rate was set to 0.001. The AAE had a symmetric architecture with three layers of encoder and decoder. The size of the hidden states for the encoder and decoder was 30. The value 30 is experimentally obtained from a series of experiments. We applied Leaky-ReLU activation to every layer, excluding the last layer.

## 4. Experimental Results

### 4.1. Experimental Setup

**Figure 4:**
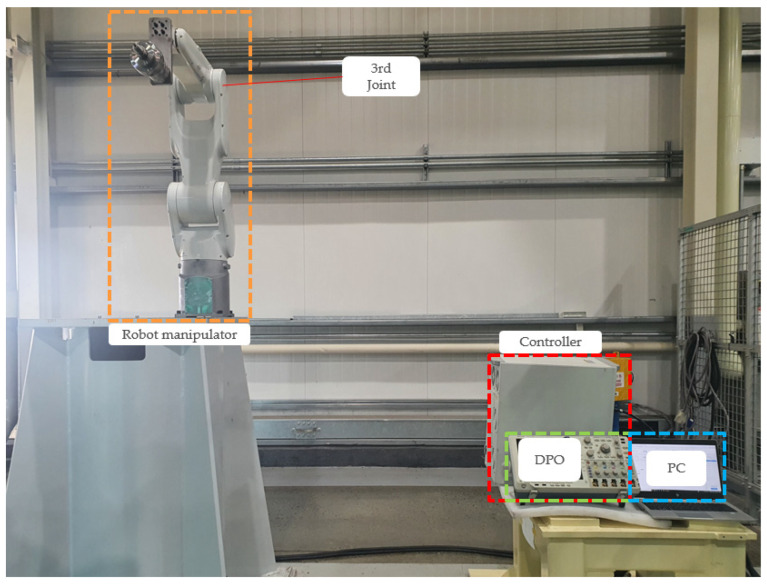
Experimental setup for the HYUNDAI HH7 manipulator and Hi5a-T10 controller.

**Figure 5:**
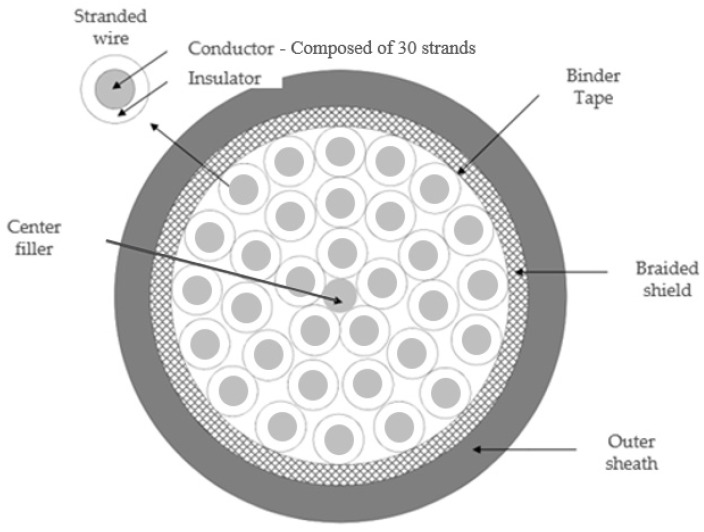
Cross sectional view of C&I cable (COVV-SB 32C × 1.5SQ).

The experiments were conducted on a HYUNDAI HH7 robot manipulator to demonstrate the effectiveness of the proposed fault-diagnosis method; the experimental setup is shown in Figure 4. The target cable was the motor control cable of the third joint of the manipulator. The Hi5a-T10 controller was used to control the motor of the joint. The specifications of the control cable are listed in Table 2. The cable has 32 wires; each wire is composed of 30 strands, and each strand has a diameter of 0.25 mm, as shown in Figure 5. The three stranded wires, i.e., three-phase, were selected out of the 32 wires. HYUNDAI HR-basic language is used to program the robot. The programming method was teaching and playback of robot manipulator using a hand-held device. The robot was programmed to move up and down by TP530 teaching device using HR-basic. A cylindrical shaped weight of 7 kg was installed on the end of the robot arm to simulate full load capacity of the robot because the robot has a maximum payload of 7 kg. The currents in phases a, b, and c were measured using a Tektronix DPO 4104 B phosphor oscilloscope at a sampling rate of 100 kHz. Even though the current sensors are already installed inside the controller for manipulator control, we used the oscilloscope for convenience.

**Figure 6:**
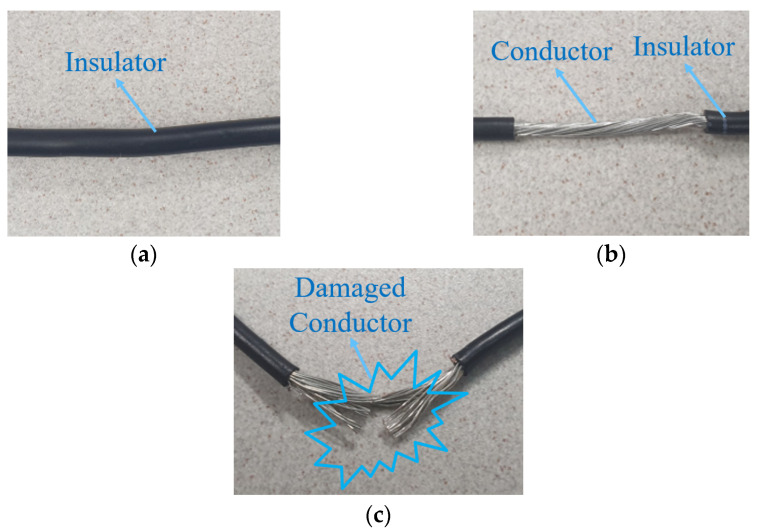
The process of inducing damage into the wires: (**a**) Healthy wire inside the cable; (**b**) Insulator-removed wire; (**c**) Faulty wire with a damaged conductor (some strands are cut).

To simulate the damage with varying severity to the wire, we artificially increased the wire’s resistance gradually by decreasing the cross-sectional area of the faulty wire. Figure 6 depicts the process of inducing damage into the wires. Figure 6a depicts the intact and healthy wire. To simulate damage, the insulator of the wire was removed, as shown in Figure 6b. A total of thirty wire strands were gradually removed to reduce the cross-sectional area of the wire. Four strands were iteratively cut per experiment to increase *S_f_* and decrease α, as shown in Figure 6c, resulting in the decrease of α from 1.000 to 0.066. To simulate faulty cable cases, artificial soft faults were induced in the phase *a* wire with varying severities (0.066 < α < 1.000). The fault length is fixed for the experiments to keep the various levels of damage under control [17].

**Figure 7:**
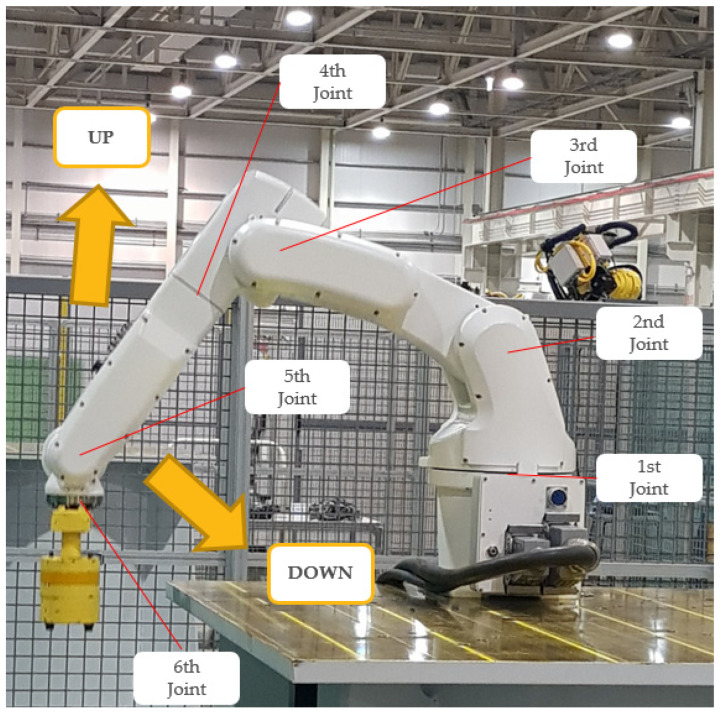
The manipulator is moving up and down for the experiment.

**Figure 8:**
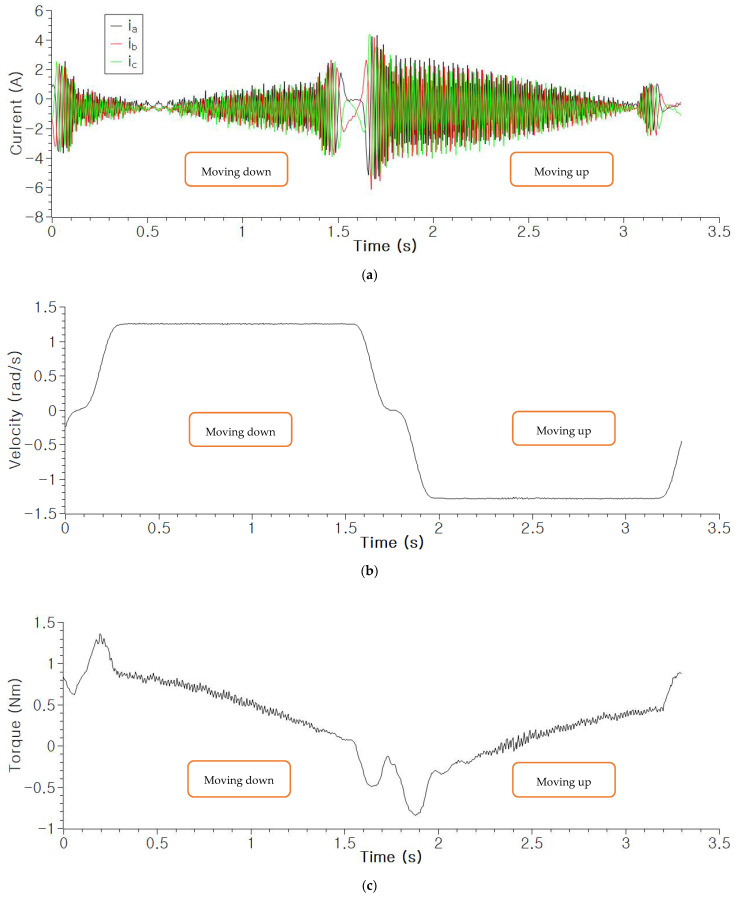
The waveforms of the three-phase currents measured from the moving manipulator (down and up). The measured signal having varying frequency (speed), phase sequence (rotational direction), and current amplitude (torque): (**a**) Measured three-phase currents (down and up); (**b**) Change in the rotational speed and direction calculated from (**a**); (**c**) Change in the torque calculated from (**a**).

To simulate the fault conditions with varying rotational speed, rotational direction, and load torque, the third joint of the manipulator was controlled to move up and down repeatedly, as shown in Figure 7. The motor in that joint is controlled by the controller to move the manipulator to the two commanded positions exactly. Throughout the process of motor control, the frequency, amplitude, and phase of the three-phase currents vary, as shown in Figure 8. The waveforms of the currents vary according to the movement of the robot joint. It can be noticed in Figure 8 that the velocity and torque vary continuously as the third joint and its arm move along the determined trajectory. Using these data, we demonstrate that the proposed diagnosis method works even under this varying condition.

The cable conditions were divided into eight classes according to the left cross-sectional area *S_f_* of the target wire, as shown in Table 1. For the experiments, a healthy target cable is prepared and damaged gradually to collect normal and fault datasets. 5000 selected windows per fault-severity class from the measured three-phase currents were utilized as training, validation, and testing datasets. The collected datasets for each severity are shown in Table 3. Each dataset is produced by windowing the measured currents and the length of the window was 9000. The 0 cut dataset, i.e., healthy cable, were used for training (3000 samples), validation (1000 samples), and testing (1000 samples) under the normal condition. The 4 cut–28 cut datasets were used as novelty testing dataset (1000 samples). For example, the 4 cut is a slight fault case with a large α compared to that of the other data; whereas, the 28 cut is a severe fault case with a small α, where only 2 strands are left out of the 30 strands owing to the harsh environment.

In addition to the eight classes corresponding to one normal condition and seven faulty conditions, one more fault condition was tested as an extra experiment. Mostly, the conductor of a wire is damaged after the insulation layer covering the conductor is damaged by the surrounding environment. Therefore, the damage of insulator also needs to be diagnosed using the proposed algorithm. From the equivalent model shown in Figure 1, the damage of insulator changes *R_i_* and *C_i_* values, resulting in the changes in currents *i_res_* and *i_cap_*, respectively. The proposed method could diagnose this type of soft fault because the method conducts the diagnosis based on the current flowing through the target cable. 

### 4.2. Results and Analysis

We tested the 11 scenarios listed in Table 4 to demonstrate the proposed method under varying operating and fault conditions. Scenarios 1–7 were prepared for each damage class, that is, 4 cut–28 cut, to be experimented individually. 4 cut class is the slightly damaged dataset, which means that in this class only 13.3 % of the conductor is damaged. The most severe damage class is 28-cut class, which means in this class that 93.3 % of the conductor is damaged; however, the damaged wire is still working without indicating any fault symptom. It can transform into a hard fault, i.e., open-circuit, within a short time, and the robot and manufacturing line can stop, resulting in unwanted down time and loss of productivity. Scenario 8 is to test for relatively slight damage classes, that is, 4 cut–12 cut, in which more than a half of the cross-sectional area of the conductor is intact, whereas, scenario 9 is to test for heavily damaged classes, that is, 16 cut–28 cut, in which less than a half of the cross-sectional area of the conductor is intact. Scenario 10 is to check if the proposed method diagnoses the damage in the insulator, while the conductor beneath the insulation layer is intact. According to the equivalent model, the proposed algorithm could diagnose it theoretically. The last scenario is to test the whole dataset at once to validate the overall diagnosis capability of the proposed algorithm.

The whole dataset was acquired from the moving robot manipulator while the third joint of the manipulator was moving up and down, as shown in Figure 7. The dataset was acquired to demonstrate that the proposed method works successfully even under varying speed, phase, and torque, as shown in Figure 8.

Table 5 presents the AUROC of the standard AAE and the improved AAE with RaPP. The best score for each scenario is underlined. The improved AAE provided good results for most of the scenarios, except for scenario 8. The highest improvement was observed in scenario 5 in that the difference of AUROC between standard and RaPP AAE was over 20 %. For the slight damage classes (scenario 8), the standard AAE detected the damage better than the AAE with RaPP. Nevertheless, the AUROCs for both the methods were greater than 0.900, which is sufficiently high for diagnosing soft faults because they tend to be transformed into hard faults gradually from slight to severe fault in chronological order. Therefore, the ongoing soft-fault development can be detected in the process of development. Moreover, the AUROC in scenario 10 was calculated to be 0.980, which is sufficiently high to confirm that the proposed method is applicable for the diagnosis of insulator damage. The equivalent circuit presented in Figure 1 worked as expected; hence, the proposed method can be used for the diagnosis of a damaged insulation layer. The scenario 11 is tested for all kind of damage classes, which are found in the soft fault developing process. Thus, this experimental result demonstrates that the proposed diagnosis model is applicable to practical problems.

The results also prove that the proposed method is applicable to varying operating conditions, such as changing rotational direction, rotational speed, and torque (Figure 8). It can be observed that the novelties can be detected using the proposed method even in the transient conditions in which the rotational direction and speed change. In addition, even if the torque changes, the faults with various degrees of severity can be detected. This is because the inputs, such as the sum of the three phase currents and the ratio of phase current to the total current, are invariant to the changes in the operating conditions but only reflect the asymmetry in the motor control network, which results in the fluctuations in the input features ($i_{sum}$, *P_j_*). These fluctuations can be recognized as novelty. Therefore, the fault detection and clear distinction between the healthy and faulty conditions are achieved. 

**Figure 9:**
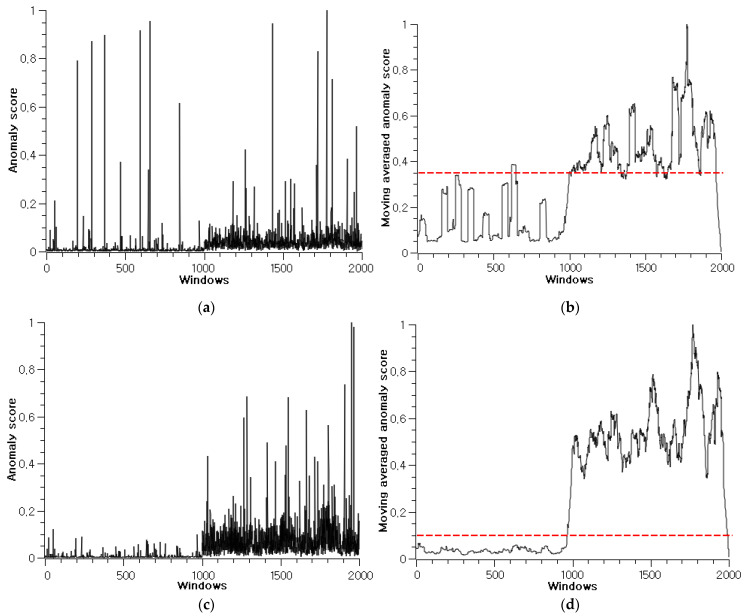
The anomaly score of scenario 11 for healthy condition (0–1000) and faulty condition (1001–2000) windows. The score shows low values for the healthy condition until the 1000th window, and the values become high for the unhealthy windows after the 1000th window. The red dashed line represents the threshold limit: (**a**) Score of standard AAE; (**b**) Moving-averaged score of standard AAE; (**c**) Score of RaPP AAE; (**d**) Moving averaged score of RaPP AAE.

The anomaly score alone is difficult for use in cable diagnosis because the score can be affected by measurement errors, cable re-routing, and environmental disturbances, making it difficult to assess the condition of the target cable, as shown in Figure 9a,c, which depicts the anomaly score graph of scenario 11. Therefore, a moving-average filter with a moving window length of 0.4 ms was used for stable diagnosis. The moving-averaged anomaly score was utilized as a fault indicator in this study. Moreover, to avoid false maintenance calls and set the diagnostic sensitivity, the threshold limit was set for the moving averaged anomaly score, as shown in Figure 9b,d. The threshold value is obtained experimentally by observing the moving averaged anomaly score of the healthy cable. The threshold limit for AAE without RaPP scoring technique needs to be set higher than that for AAE with RaPP because of its low detection accuracy. The false positive (FP), false negative (FN), true positive (TP), and true negative (TN) for Figure 9b are counted as 29, 111, 889, and 971. For Figure 9d, the values are 33, 7, 993, and 967. Using these value, the calculated accuracy and sensitivity are 0.93 and 0.89 for Figure 9b, and 0.98 and 0.99 for Figure 9d respectively. Therefore, it is demonstrated that RaPP scoring technique helps to improve the detection accuracy. In addition, the threshold value can be set to tune the sensitivity, which is related to TP and FN; sensitivity can be increased by increasing TP or by decreasing FN. By introducing the threshold, the proposed method ensures that the diagnosis method is applicable to practical cable maintenance in factories by adjusting the diagnostic sensitivity to reflect one’s maintenance standard, such as a schedule of installed cable inspection and asset management plan for old-cable replacement, according to the tendency of anomaly score graph compared with the preset threshold.

**Figure 10:**
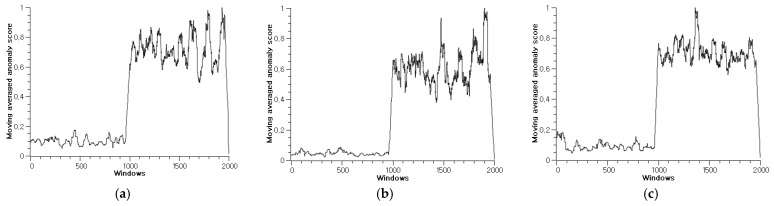

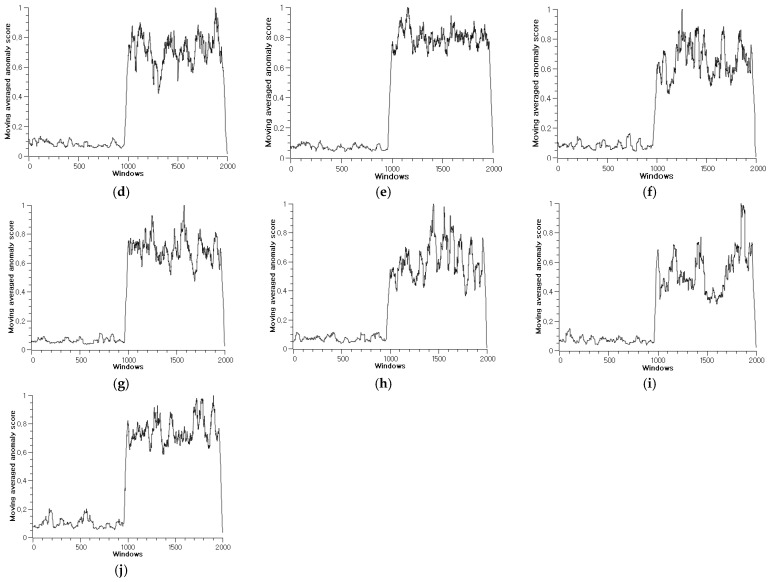
The moving-averaged anomaly score graphs of AAE with RaPP for scenarios 1–10: (**a**) Scenario 1; (**b**) Scenario 2; (**c**) Scenario 3; (**d**) Scenario 4; (**e**) Scenario 5; (**f**) Scenario 6; (**g**) Scenario 7; (**h**) Scenario 8; (**i**) Scenario 9; (**j**) Scenario 10.

According to the structure of the diagnosis model in Figure 2, the monitoring module checks the score of novelties from each window by saving the score and applying the moving-average filter. If required, the monitoring module makes a maintenance call when the moving-averaged novelty score over time exceeds the threshold limit. The moving-averaged score values increase rapidly for a damaged wire and are low and stable for a healthy wire. The score for a healthy wire is under the threshold limit. Figure 10 depicts the test results for scenarios 1–10. The moving-averaged anomaly scores for all the scenarios demonstrate that various faults can be diagnosed properly, and the isolation of faults is possible by presetting an adequate threshold limit. Moreover, these results are meaningful because only normal operation dataset was used to train the anomaly- detection module. The faults that were intentionally induced, as shown in Table 4, were properly diagnosed, and this fact make it possible to anticipate that the other indefinite kinds of soft faults, which are defined by combinations of fault length and cross-sectional area of a conductor, can also be diagnosed using the proposed method. This approach is useful if the fault dataset is very small and imbalanced, which is a usual situation in the industrial field. Unlike hard faults, that is, open-circuit and short-circuit, soft faults are difficult to be diagnosed because these faults are difficult to be detected by maintenance staff until they transform into hard faults. In many cases, a cable with soft fault continues to work, without any fault symptoms. Existing studies based on simulated faults with variable resistors or computer simulations have found implementation for this problem difficult because their methods have focused on fault conditions. In this sense, we demonstrate that the proposed diagnosis method is applicable to real world problems.

**Figure 11:**
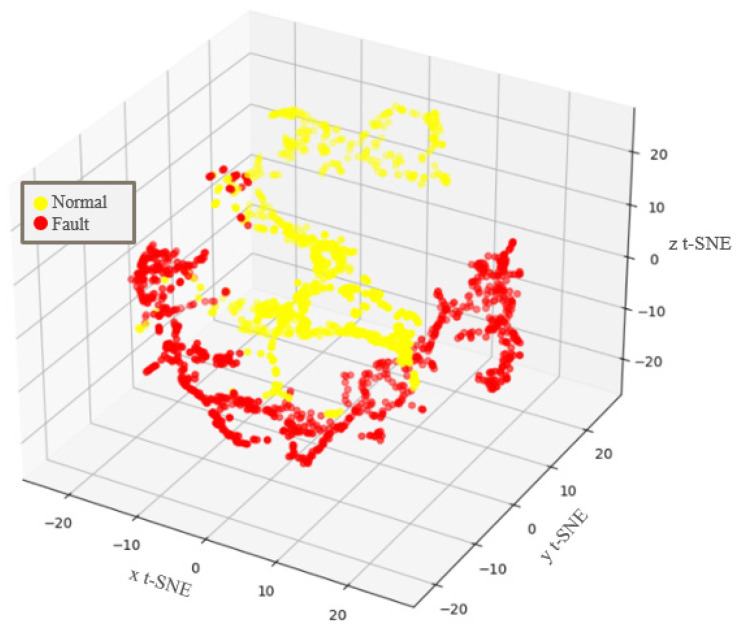
3D t-SNE feature visualization result. The axes of t-SNE define a 3D space into which the higher dimensional space will be projected, preserving relative proportional distances.

Furthermore, t-distributed stochastic neighbor embedding was used to visualize the learned high-dimensional latent space representation in terms of 3D feature vectors to verify the feature extraction capability of the proposed method [29]. The visualization of scenario 11 is shown in Figure 11. It can be observed that healthy and faulty conditions are clustered well. The healthy class, that is, 0-cut dataset, is set only to be a normal class as per Table 1 because unimodal normality is assumed to be considering one healthy class for normality. Thus, the other classes, that is, 4 cut–28 cut, and the class corresponding to a damaged insulator, belong to the faulty classes, as indicated by the red dots in Figure 11. Moreover, the results demonstrate that the proposed diagnosis method is applicable to practical problems in factory fields with changing operating conditions in that the latent space described in Figure 11 is achieved under various operation and fault conditions, such as varying speed, varying torque, varying rotational direction, and various degrees of fault severity.

### 4.3. Comparison with Other Methods

To evaluate the applicability of the proposed method, qualitative comparison with other methods were conducted, as shown in Table 6. Consequently, several indices, such as diagnosis capability, need of prior domain knowledge and testing equipment, and the application diagnosis methods, were used.

First, the mechanical techniques tend to be intrusive and destructive, and specialized training is required to operate the test equipment. Moreover, chemical techniques can be used to calculate the life expectancy of the target cable by testing. However, a part of the cable is needed to conduct the test in a laboratory. Taking the test sample can be destructive and is often not acceptable. On the other hand, electrical techniques are widely used for cable condition assessment. However, the associated test, such as partial discharge (PD), is susceptible to noise and is possible only if the cable is disconnected from the automated machine. Moreover, advanced skill set is needed to perform the analysis of the data from the test.

Reflectometry-based methods [10,11,13,14] need to consider proper compensation of the response to the incident signal because the reflected signal cannot avoid attenuation and dispersion in the cable. In addition, the issue of blind spots needs to be addressed because the incident and reflected signals may overlap for excessively short cables. More importantly, reflectometry-based methods require arbitrary waveform generators to inject the designed reference signal into the target cable.

There is research utilizing current signals to diagnose faults [16], preferred over the methods that require both current and voltage signals. The proposed method in [16] can successfully handle different kinds of power network and diagnose faults in a very short time, 1.7 ms, with a high degree of accuracy. However, the method is assuming that the amplitude and phase angles are almost equal and constant in normal operation. Unlike power plants, the currents of automated machines are varying in amplitude, frequency, and phase to control the machines. Therefore, this approach is difficult to be applied in factory automation.

The major differences between the existing methods and proposed method are as follows: (1) The proposed method does not need manual feature extraction and prior domain knowledge, such as physical parameters of cables. (2) It does not need additional equipment (cost effective); (3) It can diagnose very short cables. (4) It can be used to perform online diagnosis of the operating cable even if the operating conditions change continuously. The proposed method is applicable in the field of factory automation in which various operating conditions and different types of cables are present.

## 5. Conclusions

In this study, a novel soft-fault diagnosis scheme is proposed to diagnose faults in C&I cables using only three-phase currents. The results confirm that faults can be detected with a 98% accuracy. The proposed deep AAE consists of an encoder and a decoder with a RaPP scoring technique. The AAE structure was used to detect novelties in the inputs using the information in hidden spaces. The detecting and monitoring modules check the detected fault and generate a maintenance call when the threshold is exceeded.

The proposed diagnosis model is cost efficient because extra sensors and signal generators are not required for its application. In addition, physical parameters of cables and related information are not required. Experiments conducted on an industrial robot demonstrated that the proposed diagnostic model effectively diagnoses healthy and faulty conditions and is applicable to the automated production lines, which operate under varying and harsh environments. 

This study is focused on the diagnosis of soft faults in C&I cables of industrial machines. Localizing faults is not considered in this paper because cables from automated factories are relatively short and fixed according to factory layout. In this sense, short faulty cables tend to be replaced first and analyzed later in a case of emergency, unlike the cables from power plants where the length of cables is in the range of kilometers can be fixed after finding the faulty location. Therefore, timely diagnosis of soft faults is more important than localizing the faults in factory automation. Moreover, hard faults, such as open-circuit and short-circuit, are not considered for this study because these faults can be directly detected by machine controllers and related error codes will be generated automatically by the controllers, such as ‘Power loss in the first servo motor’, nowadays. Therefore, hard faults can be diagnosed considering the generated error codes. In addition, diagnosing soft faults in advance is more beneficial than hard faults later because soft faults have a high probability of transforming into hard faults, and a single hard fault in C&I cables could result in production line stoppages.

In future studies, we will focus on the diagnosis of soft faults in the flexible C&I cables for moving (bending, torsion) applications by combining various signals, such as voltage, temperature, and current.

## Figures and Tables

**Table 1:** Degree of damage severity.

Number of Strands Cut	Degree of Conductor Damage	
	In Percent (%)	In α
0 (normal)	0%	1
4	13.3%	0.866
8	26.6%	0.733
12	40.0%	0.600
16	53.3%	0.466
20	66.6%	0.333
24	80.0%	0.200
28	93.3%	0.066

**Table 2:** Specification of the control cable.

Subject	Unit	Specification
Number of wire	-	32
Cross-sectional area of wire	mm^2^	1.5
Number of strands per wire	-	30
Cross-sectional area of strands	mm^2^	0.25
Maximum resistance of wire	Ω/km	17.7
Insulator	-	PVC
Insulator thickness	-	0.36
Shield type	-	Braided
Diameter	mm	20.4

**Table 3:** Total dataset for each damage severity.

Damage Class	Damage Severity (α)	Number of Dataset
0 cut (normal)	1	5000
4 cuts	0.866	5000
8 cuts	0.733	5000
12 cuts	0.600	5000
16 cuts	0.466	5000
20 cuts	0.333	5000
24 cuts	0.200	5000
28 cuts	0.066	5000

**Table 4:** Scenarios for experiment.

Number of Scenario	Damage Classes	Robot Movement
Scenario 1	Normal, 4 cut	up and down
Scenario 2	Normal, 8 cut	up and down
Scenario 3	Normal, 12 cut	up and down
Scenario 4	Normal, 16 cut	up and down
Scenario 5	Normal, 20 cut	up and down
Scenario 6	Normal, 24 cut	up and down
Scenario 7	Normal, 28 cut	up and down
Scenario 8	Normal, 4–12 cut	up and down
Scenario 9	Normal, 16–28 cut	up and down
Scenario 10	Normal, insulator damaged	up and down
Scenario 11	Normal, 4–28 cut, insulator damaged	up and down

**Table 5:** AUROC with novelty datasets.

Scenarios	AAE	
	Standard	With RaPP
Scenario 1	0.933	0.949
Scenario 2	0.959	0.971
Scenario 3	0.956	0.967
Scenario 4	0.900	0.980
Scenario 5	0.804	0.971
Scenario 6	0.899	0.943
Scenario 7	0.798	0.916
Scenario 8	0.972	0.945
Scenario 9	0.948	0.968
Scenario 10	0.921	0.980
Scenario 11	0.945	0.981

**Table 6:** Comparison with other methods.

	Proposed Method	Mechanical (EAB)	Chemical (OIT)	Electrical (TD, PD)	[10]	[11]	[13]	[14]	[16]
Applicability to very short cables	O ^1^	O	O	O	X	X	X	X	O
Online diagnosis under varying conditions (*I_m_*, *ω*, *ϕ*)	O	X	X	X	X	X	X	O	X
Nonintrusive and nondestructive	O	X	X	O	O	O	O	O	O
Require prior domain knowledge	X ^1^	O	O	O	O	O	O	O	X
Require waveform generator	X	X	X	X	O	O	O	O	X
Require dedicated equipment	X	O	O	O	X	X	X	X	X

^1^ For the first three, O is better. For the last three, X is better.

## Data Availability

The data presented in this study are available on request from the corresponding author. The data are not publicly available because it is company confidential information.
